# The structural balance analysis of complex dynamical networks based on nodes' dynamical couplings

**DOI:** 10.1371/journal.pone.0191941

**Published:** 2018-01-31

**Authors:** Zilin Gao, Yinhe Wang

**Affiliations:** 1 School of Automation, Guangdong University of Technology, Guangzhou, Guangdong Province, China; 2 School of Computer Science and Engineering, Chongqing Three Gorges University, Chongqing, China; Lanzhou University of Technology, CHINA

## Abstract

The nodes and their connection relationships are the two main bodies for dynamic complex networks. In existing theoretical researches, the phenomena of stabilization and synchronization for complex dynamical networks are generally regarded as the dynamic characteristic behaviors of the nodes, which are mainly caused by coupling effect of connection relationships between nodes. However, the connection relationships between nodes are also one main body of a time-varying dynamic complex network, and thus they may evolve with time and maybe show certain characteristic phenomena. For example, the structural balance in the social networks and the synaptic facilitation in the biological neural networks. Therefore, it is important to investigate theoretically the reasons in dynamics for the occurrence. Especially, from the angle of large-scale systems, how the dynamic behaviors of nodes (such as the individuals, neurons) contribute to the connection relationships is one of worthy research directions. In this paper, according to the structural balance theory of triad proposed by F. Heider, we mainly focus on the connection relationships body, which is regarded as one of the two subsystems (another is the nodes body), and try to find the dynamic mechanism of the structural balance with the internal state behaviors of the nodes. By using the Riccati linear matrix differential equation as the dynamic model of connection relationships subsystem, it is proved under some mathematic conditions that the connection relationships subsystem is asymptotical structural balance via the effects of the coupling roles with the internal state of nodes. Finally, the simulation example is given to show the validity of the method in this paper.

## Introduction

In 1946, the structural balance concept is proposed originally by F. Heider [1] to model psychologically the dynamic characteristics of connection relationships between individuals, and has been identified experimentally in society of hyraxes [2]. Specifically, the structural balance theory begins with the equilibrium analysis of the triad, and is determined by the product of the 3 edges: if it is positive, then the triad is structural balance; otherwise, the triad is structural non-balance. In previous works on the Heider balance, the values of connection strength are described with integers 0 and ±1 [3,4]. In 2005, the continuous-valued interaction strengths are proposed in [5–8], and then some time-varying dynamic equations for interaction strengths are discussed in [9–14]. These papers investigate the time evolution behaviors of the connection relationships and show certain basic features of the long-time dynamics of connection relationships between individuals. However, these above papers did not pay attention to the effects of internal state motions of the individuals on the evolution of connection relationships.

In fact, from the angle of mathematical graph theory, the individuals and their connection relationships form the complex network described by the complete graph [15], and the nodes and the connection relationships are regarded as the two basic elements of the complex dynamical networks, they are usually coupled with each other in the evolution of the network over time, and the dynamical change of any one between the two basic elements will be transferred to the other through the effective coupling relationships. Therefore, it is worth studying how the dynamical changes of the nodes’ states affect the structural balance of connection relationships.

From the angle of large-scale system theory [16], the complex dynamical networks are composed of nodes subsystem and connection relationships subsystem. It is noticed that in the existing theoretical research results about complex dynamical networks, almost all of them show that the nodes are the main body resulting in synchronization or stabilization behaviors, and the connection relationships are regarded only as the auxiliary part due to coupling roles between the nodes. For example, the following cases are considered in [17–27]: (i) the connection relationships of the nodes are known constants. For this case, the control design methods of synchronization or stabilization with some conditions are proposed for complex dynamical networks in [17–20], which show the nodes are the main body resulting in synchronization or stabilization behaviors; (ii) the connection relationships are time varying and continuous deterministic. For this case, the synchronization or stabilization control problems are discussed with some conditions in [21–24], which show also the nodes are the main body resulting in synchronization or stabilization behaviors; (iii) the connection relationships are unknown constants. For this case, by using the parameter adaptive method, the adaptive synchronization or stabilization controllers are researched in [25–27], which show also the nodes are the main body resulting in synchronization or stabilization behavior.

It is not hard to see the existing research results about complex dynamical networks such as [17–27] only focus on the effects of connection relationships on the dynamical behaviors of the nodes, and ignore the effects of the nodes on the dynamical behaviors of the connection relationships, such as one important phenomenon called the structural balance. In other word, the dynamic characteristics of the connection relationships are not concerned in [17–27]. From the perspective of dynamics, the dynamical evolution of complex network with time means that the nodes and connection relationships do the time varying motion together with their mutual coupling. This implies that any dynamical change of nodes will cause the connection strength to be changed via the coupling relationships. For example, Gamma oscillations in neurons (nodes) may cause the synaptic facilitation (connection relationships) in the biological neural network [28,29]. In the biological community, the changes in niche breadth of species (nodes) may change the intensity of competition between them (connection relationships) [30,31]. Therefore, if the complex network is dynamically time varying evolving, similar to the synchronization or stabilization regarded as dynamic characteristics of nodes, it is of scientific significance to find the dynamic characteristics of connection relationships.

In order to use the structural balance theory in this paper, we consider a complete time-varying complex dynamical network with continuous time values for connection strengths, where the connection relationships subsystem is described by the Riccati matrix differential equation possessing the coupling matrix composed of the internal state of the nodes. In this paper, we focus on the connection relationships subsystem and want to design the coupling matrix to guarantee the structural balance to be emerged under some mathematic conditions. It is noteworthy that this paper focuses on the mechanism of generating the structural balance for the connection relationships, and the dynamical changes of the nodes’ state only play an auxiliary part by the coupling matrix. This is contrary to the existing researches on synchronization or stabilization behavior in complex dynamical networks.

This paper is organized as the following sections. In Section II, the mathematic model for complex dynamical network is proposed, which is composed of the nodes subsystem and the connection relationships subsystem with their mutually coupling. In Section III, the equilibrium state of the complex dynamical network is introduced by using the equilibrium states of the nodes subsystem and the connection relationships subsystem. In Section IV, the structural balance matrix is introduced. The coupling matrix in the mathematic model of connection relationships subsystem is proposed to ensure the structural balance to be asymptotically achieved. In Section V, the result about asymptotical structural balance is mathematically proved under some conditions. In Section VI, the illustrative example is presented to demonstrate the proposed design procedure and validity. The conclusion is given in Section VII.

## Network model description

Consider the undirected complex dynamical network with *N* nodes, in which not only the nodes are dynamically changing with time, but also the strength of the connection relationships between nodes is dynamical and continuous.

In this paper, we only consider this case that each node is one dimensional continuous time-varying system, and the dynamical equation of node *i* can be expressed as:

$${\dot{x}}_{i}=f_{i}(x_{i}),i=1,2,\cdots ,N$$ (1)

where *x*_*i*_ ∈ *R* is the state variable of node *i*, and *f*_*i*_(*x*_*i*_) is a continuous function.

The connection relationship strength between the node *i* and the node *j* in the network can be expressed by the time-varying function *p*_*ij*_(*t*), and *p*_*ij*_ = *p*_*ji*_ for undirected networks. Especially, when *i* = *j*, *p*_*ii*_ indicates the relationship strength of the node itself. Let *c* ∈ *R* be a given real number, which represents the common connection relationship strength in the network. Then the interconnected dynamical equation composed of all nodes reads as

$${\dot{x}}_{i}=f_{i}(x_{i})+c\sum_{j=1}^{N}p_{ij}(t)H_{j}(x_{j}),i=1,2,\cdots ,N$$ (2)

where *H*_*j*_(*x*_*j*_) is a continuous function.

**Remark 1.** The dynamical model (2) is a typical complex dynamical network model about the nodes, if the connection relationship *p*_*ij*_ is a constant, and $p_{ii}=-\sum_{j=1,j\neq i}^{N}p_{ij}$ is true, the system (2) is called the time-invariant dissipative coupled complex dynamical network and shown in [18–20,32]; If the connection relationship *p*_*ij*_ is time-varying, the system (2) is called time-varying complex dynamical network and shown in [21–24,33].

The matrix composed of the connection relationships strengths in the network (2) can be expressed by *P* = *P*(*t*) = (*p*_*ij*_(*t*))_*N*×*N*_ ∈ *R*^*N*×*N*^.

Let the state vector *x* = [*x*_1_ *x*_2_ ⋯ *x*_*N*_]^*T*^ ∈*W*, where *W* is a compact set in *R*^*N*^. *f*(*x*) = [*f*_1_(*x*_1_) *f*_2_(*x*_2_) ⋯ *f*_*N*_(*x*_*N*_)]^*T*^ and *H*(*x*) = [*H*_1_(*x*_1_) *H*_2_(*x*_2_) ⋯ *H*_*N*_(*x*_*N*_)]^*T*^ are continuous vector functions, then the dynamical Eq (2) can be described by

$$\dot{x}=f(x)+cP(t)H(x)$$ (3)

where the connection relationships matrix *P* = *P*(*t*) satisfies the Riccati dynamical equation as follows

$$\dot{P}=AP+PA^{T}+\Phi (x)$$ (4)

where the matrix *A* ∈ *R*^*N*×*N*^, and *Φ*(*x*) ∈ *R*^*N*×*N*^ represents the coupling matrix with the internal state of nodes.

**Remark 2.** (i) When the fully connected networks with constant connection relationships are considered, the connection relationships matrix *P* is a constant matrix, that is $\dot{P}=0$, so the constant matrix *P* can be regarded as a special case of the dynamical Riccati Eq (4). (ii) It is easily seen that the nodes subsystem (3) and the connection relationships subsystem (4) are mutually coupling, and the dynamical behavior of the connection relationships (4) is related to the form of the coupling matrix *Φ*(*x*), moreover, the coupling matrix *Φ*(*x*) is determined by the nodes’ state. Therefore, this paper mainly focuses on the coupling matrix *Φ*(*x*) by which the nodes’ state will force the connection relationships matrix *P* in (4) asymptotically to achieve structural balance.

**Assumption 1.** The vector function *H*(*x*) is bounded, that is, there exists a known positive number *h* satisfying the inequality ‖*H*(*x*)‖≤*h*.

## Equilibrium state of the network

The equilibrium state of the complex dynamical network is composed of the ones of the subsystems (3) and (4), which is denoted as {x*, P*} and satisfies the following algebraic equations:

$$f(x^{*})+cP^{*}H(x^{*})=0$$ (5)

$$AP^{*}+P^{*}A^{T}+\Phi (x^{*})=0$$ (6)

where the equilibrium vector $x^{*}={\left[\begin{array}{ccc} \begin{array}{cc} x_{1}^{*} & x_{2}^{*} \end{array} & \cdots & x_{N}^{*} \end{array}\right]}^{T}$, and assume $x_{i}^{*}\neq 0,i=1,2,\cdots ,N$.

It is obvious that x* is the equilibrium vector of the subsystem (3). The Eq (6) is an algebraic Ricatti equation. If the equilibrium vector x* is given and the matrix *A* ∈ *R*^*N*×*N*^ is a given Hurwitz matrix, through the literatures [34,35], we can get the equilibrium matrix of the subsystem (4) is

$$P^{*}=-\int_{0}^{+\infty }e^{At}\Phi (x^{*})e^{A^{T}t}dt$$ (7)

## The structural balance of the network

For undirected networks, the concept of the structural balance was proposed originally by F. Heider [1]. The connection relationships matrix *P* was described by symmetric matrix in [5,6,11,12], and the diagonal elements of the symmetric matrix are positive and indicate the self-identity and the strength of confidence under the sociological sense in [12]. In this paper, the definition of the structural balance matrix is given as follows.

**Definition 1.** If the elements of the real symmetric matrix *S* = (*s*_*αβ*_) ∈ *R*^*n*×*n*^ satisfy the inequality *s*_*αβ*_*s*_*βρ*_*s*_*ρα*_ > 0, for *α*,*β*,*ρ* = 1,2,⋯,*n*, where *n* ≥ 3, then the symmetric matrix *S* is called the structural balance matrix.

According to the Definition 1, the asymptotical structural balance of the complex dynamical network can be defined as follows.

**Definition 2.** Consider the complex dynamical network composed of the subsystems (3) and (4), and its equilibrium state satisfies the Eqs (5) and (6). The equilibrium matrix P* is a structural balance matrix with the algebraic Ricatti Eq (6). If $P(t)\overset{t\to +\infty }{\to }P^{*}$, then the complex dynamical network composed of the subsystems (3) and (4) is called as asymptotical structural balance.

**Remark** 3. (i) From Definition 2, we know that the fully connected network with the positive constant connection relationships is asymptotical structural balance; this kind of networks can be called as the trivial asymptotical structural balance. (ii) Obviously, if the Eqs (5) and (6) are true, the structural balance of the matrix P* is directly related to the specific form of the coupling matrix *Φ*(*x*) ∈ *R*^*N*×*N*^. In this paper, we consider the following form of *Φ*(*x*).

$$\Phi (x)=-A(xy^{T}+yx^{T})-(xy^{T}+yx^{T})A$$ (8)

where *y* is proposed as the following two forms:

(i)y=x (9)

(ii) We choose a constant vector *y* = [*y*_1_ *y*_2_ ⋯ *y*_*N*_]^*T*^ in which the sign of each element is same as the equilibrium vector x* of the subsystem (3), that is, *y*_*i*_ satisfies the following inequality

$$y_{i}x_{i}^{*}>0,i=1,2,\cdots ,N$$ (10)

**Lemma 1.** Consider the complex dynamical network composed of subsystems (3) and (4), and the Eqs (5) and (6) hold. If the coupling matrix *Φ*(*x*) is as shown in (8), then the equilibrium matrix P* is structural balance.

**Proof.** The following two cases are used to prove the Lemma 1.

**Case 1.** *y* = *x*

In this case, the Eqs (6) may be changed to

$$AP^{*}+P^{*}A^{T}+\Phi (x^{*})=AP^{*}+P^{*}A^{T}-2A\left[x^{*}{\left(x^{*}\right)}^{T}\right]-2\left[x^{*}{\left(x^{*}\right)}^{T}\right]A=0$$

Obviously, P* = 2x*(x*)^*T*^ is the solution of the above equation, and we easily obtain

$$p_{ij}^{*}p_{jk}^{*}p_{ki}^{*}=\left(2x_{i}^{*}x_{j}^{*}\right)\left(2x_{j}^{*}x_{k}^{*}\right)\left(2x_{k}^{*}x_{i}^{*}\right)=8{\left(x_{i}^{*}x_{j}^{*}x_{k}^{*}\right)}^{2}>0$$ (11)

According to the Definition 1 and $x_{i}^{*}\neq 0,i=1,2,\cdots ,N$, we know that the matrix P* = 2x*(x*)^*T*^ is structural balance.

**Case 2.** The constant vector *y* = [*y*_1_ *y*_2_ ⋯ *y*_*N*_]^*T*^ and $y_{i}x_{i}^{*}>0,i=1,2,\cdots ,N$.

In this case, it is easy to see that the solution of the Eqs (6) is P* = x*y^*T*^ + *y*(x*)^*T*^. Hence, we can directly calculate and obtain

$$\begin{array}{l} p_{ij}^{*}p_{jk}^{*}p_{ki}^{*}=(x_{i}^{*}y_{j}+y_{i}x_{j}^{*})(x_{j}^{*}y_{k}+y_{j}x_{k}^{*})(x_{k}^{*}y_{i}+y_{k}x_{i}^{*})\\ \;=(x_{i}^{*}y_{j}x_{j}^{*}y_{k}+x_{i}^{*}y_{j}y_{j}x_{k}^{*}+y_{i}x_{j}^{*}x_{j}^{*}y_{k}+y_{i}x_{j}^{*}y_{j}x_{k}^{*})(x_{k}^{*}y_{i}+y_{k}x_{i}^{*})\\ \;=(x_{i}^{*}y_{i})(y_{j}x_{j}^{*})(y_{k}x_{k}^{*})+(x_{i}^{*}y_{i})y_{j}y_{j}x_{k}^{*}x_{k}^{*}+(y_{k}x_{k}^{*})y_{i}y_{i}x_{j}^{*}x_{j}^{*}\\ \;+(x_{j}^{*}y_{j})x_{k}^{*}x_{k}^{*}y_{i}y_{i}+(y_{j}x_{j}^{*})y_{k}y_{k}x_{i}^{*}x_{i}^{*}+(x_{k}^{*}y_{k})x_{i}^{*}x_{i}^{*}y_{j}y_{j}\\ \;+(y_{i}x_{i}^{*})x_{j}^{*}x_{j}^{*}y_{k}y_{k}+(x_{j}^{*}y_{j})(x_{k}^{*}y_{k})(x_{i}^{*}y_{i})\\ \;=(x_{i}^{*}y_{i})(y_{j}x_{j}^{*})(y_{k}x_{k}^{*})+(x_{i}^{*}y_{i}){\left(x_{k}^{*}\right)}^{2}y_{j}^{2}+(y_{k}x_{k}^{*}){\left(x_{j}^{*}\right)}^{2}y_{i}^{2}+(x_{j}^{*}y_{j}){\left(x_{k}^{*}\right)}^{2}y_{i}^{2}+\\ \;(y_{j}x_{j}^{*}){\left(x_{i}^{*}\right)}^{2}y_{k}^{2}+(x_{k}^{*}y_{k}){\left(x_{i}^{*}\right)}^{2}y_{j}^{2}+(y_{i}x_{i}^{*}){\left(x_{j}^{*}\right)}^{2}y_{k}^{2}+(x_{j}^{*}y_{j})(x_{k}^{*}y_{k})(x_{i}^{*}y_{i})\\ \;=2(x_{i}^{*}y_{i})(y_{j}x_{j}^{*})(y_{k}x_{k}^{*})+\left[(x_{i}^{*}y_{i}){\left(x_{k}^{*}\right)}^{2}+(x_{k}^{*}y_{k}){\left(x_{i}^{*}\right)}^{2}\right]y_{j}^{2}+\\ \;\left[(y_{k}x_{k}^{*}){\left(x_{j}^{*}\right)}^{2}+(x_{j}^{*}y_{j}){\left(x_{k}^{*}\right)}^{2}\right]y_{i}^{2}+\left[(y_{j}x_{j}^{*}){\left(x_{i}^{*}\right)}^{2}+(y_{i}x_{i}^{*}){\left(x_{j}^{*}\right)}^{2}\right]y_{k}^{2} \end{array}$$ (12)

Noticing $y_{i}x_{i}^{*}>0,i=1,2,\cdots ,N$, and we can obtain the following inequality from (12)

$$p_{ij}^{*}p_{jk}^{*}p_{ki}^{*}>0$$ (13)

Finally, Case 1 and Case 2 complete the proof of Lemma 1.

## The asymptotical structural balance analysis of the network

Now, we introduce the vec(⋅) operator in [35] which maps an *m* ×*n* matrix *B* = (*b*_*ij*_) onto the vector composed of the columns of *B*

$$\mathrm{vec}(B)={\left(\begin{array}{llllllllll} b_{11}, & \cdots , & b_{m1}, & b_{12}, & \cdots , & b_{m2}, & \cdots , & b_{1n}, & \cdots , & b_{mn} \end{array}\right)}^{T}$$ (14)

Let us introduce the defining of Kronecker product for matrices.

**Definition 3** [35]. If *B* ∈ *R*^*m*×*n*^, *D* ∈ *R*^*g*×*d*^, then the Kronecker product of *B* and *D*, noted as *B* ⊗ *D* ∈ *R*^*mg*×*nd*^, is defined by the matrix

$$B⊗D=\left(\begin{array}{llll} b_{11}D & b_{12}D & \cdots & b_{1n}D\\ b_{21}D & b_{22}D & \cdots & b_{2n}D\\ \cdot & \cdot & \cdot & \cdot \\ \cdot & \cdot & \cdot & \cdot \\ b_{m1}D & b_{m2}D & \cdots & b_{mn}D \end{array}\right)$$ (15)

In this paper, the following results are given [35]:

- vec(*BXD*) = (*B* ⊗ *D*^*T*^)vec(*X*);
- vec(*BX* + *XD*) = (*B* ⊗ *I* + *I* ⊗ *D*^*T*^)vec(*X*).

For simplicity, the following notations are used. $\bar{x}=x-x^{*}$, $\bar{P}=P-P^{*}$ and $\bar{\Phi }=\Phi (x)-\Phi (x^{*})$ denote the errors for corresponding variables, respectively. It is easily to verify the errors $\bar{x}$ and $\bar{P}$ satisfy the following dynamic differential equation by using (3)-(6), respectively.

$$\dot{\bar{x}}=f(x)-f(x^{*})+c[P(t)H(x)-P^{*}H(x^{*})]$$ (16)

$$\dot{\bar{P}}=A\bar{P}+\bar{P}A^{T}+\bar{\Phi }$$ (17)

By (13), (14) and the properties of Kronecker, the dynamic differential Eq (17) can be transformed to

$$\mathrm{vec}(\dot{\bar{P}})=\mathrm{vec}(A\bar{P}+\bar{P}A^{T})+\mathrm{vec}(\bar{\Phi })=(A⊗I+I⊗A)\mathrm{vec}(\bar{P})+\mathrm{vec}(\bar{\Phi })$$ (18)

For simplicity, let $\tilde{A}=A⊗I+I⊗A$.

**Assumption 2.** The vector function *H*(*x*) satisfies Lipschitz condition on a compact set *W*, that is, there exists a positive constant *δ* such that $\left\|H(x)-H(x^{*})\right\|\leq \delta \left\|\bar{x}\right\|$ for *x*,x* ∈ *W*.

**Remark 4.** If $\frac{\partial H_{j}(x_{j})}{\partial x_{j}}$, *j* = 1,2,⋯, *N*, are continuous on *W*, then Lipschitz constant *δ* in Assumption 2 may be regarded as $\delta =\underset{x\in W}{\sup}\left\|{\left(\frac{\partial H(x)}{\partial x^{T}}\right)}_{N\times N}\right\|$, where ‖•‖ expresses the Euclidean norm of a matrix.

**Assumption 3.** The vector $\mathrm{vec}(\Phi (x))=\tilde{A}\mathrm{vec}(xy^{T}+yx^{T})=\tilde{A}[\phi_{11}(x),\cdots ,\phi_{ij}(x),$ ⋯,*ϕ*_*NN*_(*x*)]^*T*^, where *ϕ*_*ij*_ = *x*_*i*_*y*_*j*_ + *x*_*j*_*y*_*i*_, satisfies Lipschitz condition on a compact set *x* ∈ *W*, that is, there exists a positive constant *L* such that $\left\|\mathrm{vec}(\Phi (x))-\mathrm{vec}(\Phi (x^{*}))\right\|\leq L\left\|\bar{x}\right\|$ for *x*,x* ∈ *W*.

**Remark 5.** If $\frac{\partial \phi_{ij}(x)}{\partial x_{i}}$, *i* = 1,2,⋯,*N*; *j* = 1,2,⋯,*N*, are continuous on *W*, then Lipschitz constant *L* in Assumption 3 may be regarded as $L=\underset{x\in W}{\sup}\left\|{\left(\frac{\partial \mathrm{vec}(\Phi (x))}{\partial x^{T}}\right)}_{N^{2}\times N}\right\|=\underset{x\in W}{\sup}\left\|\tilde{A}{\left(\frac{\partial \phi_{ij}}{\partial x^{T}}\right)}_{N^{2}\times N}\right\|$, and because $\left\|\tilde{A}{\left(\frac{\partial \phi_{ij}}{\partial x^{T}}\right)}_{N^{2}\times N}\right\|\leq \left\|\tilde{A}\right\|\left\|\frac{\partial \phi_{ij}(x)}{\partial x^{T}}\right\|\;=\left\|\tilde{A}\right\|\sqrt{\sum_{i=1}^{N}\left[{\left(2y_{i}\right)}^{2}+2(N-1)y_{i}^{2}\right]}$, we can choose $L=\left\|\tilde{A}\right\|\underset{y\in \bar{W}}{\sup}\sqrt{\sum_{i=1}^{N}\left[{\left(2y_{i}\right)}^{2}+2(N-1)y_{i}^{2}\right]}$.

**Assumption 4.** Consider the dynamical Eq (1). For a given positive matrix $\bar{Q}\in R^{N\times N}$, there exists one positive definite symmetric matrix *K* ∈ *R*^*N*×*N*^ such that the following Lyapunov equation is true on *W*.

$$J^{T}K+KJ=-\bar{Q}$$ (19)

where $J=\frac{\partial f(x)}{\partial x^{T}}$ is the Jacobian matrix.

**Assumption 5.** Consider the dynamical equation of the subsystem (4). The matrix *A* in (4) is Hurwitz stable, that is, all the eigenvalues of matrix *A* have negative real parts.

From Assumption 5 and the results in [35], we know matrix $\tilde{A}$ is also Hurwitz stable, therefore, there exists one positive symmetry matrix $M\in R^{N^{2}\times N^{2}}$ such that the following Lyapunov equation is true for a given positive matrix $Q\in R^{N^{2}\times N^{2}}$.

$$M\tilde{A}+{\tilde{A}}^{T}M=-Q$$ (20)

**Theorem 1.** Consider the complex dynamical network composed of subsystems (3) and (4), and its equilibrium state and the coupling matrix *Φ*(*x*) satisfy the Eqs (5), (6) and (8), respectively. If Assumption 1-Assumption 5 hold, and the inequality $\lambda_{\min}(Q)[\lambda_{\min}(\bar{Q})-2c\delta \left\|KP^{*}\right\|]-{\left(L\left\|M\right\|+hc\left\|K\right\|\right)}^{2}>0$ is true, then the complex dynamical network is asymptotical structural balance.

**Remark 6**. In Theorem 1, *λ*_min_(Q) and $\lambda_{\min}(\bar{Q})$ represent the minimum eigenvalues of given positive matrices *Q* and $\bar{Q}$, and the matrices *K* and *M* can be obtained by solving the Lyapunov Eqs (19) and (20), respectively; The given constant *c* represents the common connection relationship strength in the network dynamical model (2); The parameters *h*, *δ* and *L* can be obtained by Assumptions 1, 2 and 3, respectively; the equilibrium matrix P* can obtained by Eqs (6) and (8).

**Proof.** Consider the following positive definition function

$$V=\mathrm{vec}{\left(\bar{P}\right)}^{T}M\mathrm{vec}(\bar{P})+{\bar{x}}^{T}K\bar{x}.$$ (21)

Then the orbit derivative of *V* along (16) and (17) reads as

$$\begin{array}{l} \dot{V}=\mathrm{vec}{\left(\dot{\bar{P}}\right)}^{T}M\mathrm{vec}(\bar{P})+\mathrm{vec}{\left(\bar{P}\right)}^{T}M\mathrm{vec}(\dot{\bar{P}})+{\dot{\bar{x}}}^{T}K\bar{x}+{\bar{x}}^{T}K\dot{\bar{x}}\\ \;={\left(\tilde{A}\mathrm{vec}(\bar{P})+\mathrm{vec}(\bar{\Phi })\right)}^{T}M\mathrm{vec}(\bar{P})+\mathrm{vec}{\left(\bar{P}\right)}^{T}M(\tilde{A}\mathrm{vec}(\bar{P})+\mathrm{vec}(\bar{\Phi }))+\\ \;{\left\{f(x)-f(x^{*})+c[P(t)H(x)-P^{*}H(x^{*})]\right\}}^{T}K\bar{x}+\\ \;{\bar{x}}^{T}K\{f(x)-f(x^{*})+c[P(t)H(x)-P^{*}H(x^{*})]\}\\ \;=\mathrm{vec}{\left(\bar{P}\right)}^{T}({\tilde{A}}^{T}M+M\tilde{A})\mathrm{vec}(\bar{P})+\mathrm{vec}{\left(\bar{\Phi }\right)}^{T}M\mathrm{vec}(\bar{P})+\mathrm{vec}{\left(\bar{P}\right)}^{T}M\mathrm{vec}(\bar{\Phi })+\\ \;{\left(J\bar{x}\right)}^{T}K\bar{x}+{\bar{x}}^{T}KJ\bar{x}+2c{\bar{x}}^{T}K\{[P(t)H(x)-P^{*}H(x^{*})]\}\\ \;=-\mathrm{vec}{\left(\bar{P}\right)}^{T}Q\mathrm{vec}(\bar{P})+2\mathrm{vec}{\left(\bar{P}\right)}^{T}M\mathrm{vec}(\bar{\Phi })+{\bar{x}}^{T}J^{T}K\bar{x}+{\bar{x}}^{T}KJ\bar{x}+\\ \;2c{\bar{x}}^{T}K\{P(t)H(x)-P^{*}H(x)+P^{*}H(x)-P^{*}H(x^{*})\}\\ \;=-\mathrm{vec}{\left(\bar{P}\right)}^{T}Q\mathrm{vec}(\bar{P})+2\mathrm{vec}{\left(\bar{P}\right)}^{T}M\mathrm{vec}(\bar{\Phi })+{\bar{x}}^{T}J^{T}K\bar{x}+{\bar{x}}^{T}KJ\bar{x}+\\ \;2c{\bar{x}}^{T}K\{\bar{P}(t)H(x)+P^{*}[H(x)-H(x^{*})]\}\\ \;\leq -\mathrm{vec}{\left(\bar{P}\right)}^{T}Q\mathrm{vec}(\bar{P})+2\left\|\mathrm{vec}{\left(\bar{P}\right)}^{T}\right\|\left\|M\right\|\left\|\mathrm{vec}(\bar{\Phi })\right\|-{\bar{x}}^{T}\bar{Q}\bar{x}+\\ \;2c\left\|{\bar{x}}^{T}K\bar{P}(t)H(x)\right\|+2c\left\|{\bar{x}}^{T}KP^{*}[H(x)-H(x^{*})]\right\|\\ \;\leq -\lambda_{\min}(Q){\left\|\mathrm{vec}(\bar{P})\right\|}^{2}+2L\left\|\mathrm{vec}{\left(\bar{P}\right)}^{T}\right\|\left\|M\right\|\left\|\bar{x}\right\|-\lambda_{\min}(\bar{Q}){\left\|\bar{x}\right\|}^{2}+\\ \;2hc\left\|K\right\|\cdot \left\|\bar{x}\right\|\left\|\bar{P}\right\|+2c\delta \left\|KP^{*}\right\|\cdot {\left\|\bar{x}\right\|}^{2}\\ \;=-\lambda_{\min}(Q)({\left\|\mathrm{vec}(\bar{P})\right\|}^{2}-\frac{2L\left\|M\right\|}{\lambda_{\min}(Q)}\left\|\mathrm{vec}{\left(\bar{P}\right)}^{T}\right\|\left\|\bar{x}\right\|+\frac{\lambda_{\min}(\bar{Q})}{\lambda_{\min}(Q)}{\left\|\bar{x}\right\|}^{2})+\\ \;2hc\left\|K\right\|\cdot \left\|\bar{x}\right\|\left\|\bar{P}\right\|+2c\delta \left\|KP^{*}\right\|\cdot {\left\|\bar{x}\right\|}^{2}\\ \;=-\lambda_{\min}(Q)({\left\|\mathrm{vec}(\bar{P})\right\|}^{2}-\frac{2L\left\|M\right\|+2hc\left\|K\right\|}{\lambda_{\min}(Q)}\left\|\mathrm{vec}(\bar{P})\right\|\left\|\bar{x}\right\|+\frac{\lambda_{\min}(\bar{Q})-2c\delta \left\|KP^{*}\right\|}{\lambda_{\min}(Q)}{\left\|\bar{x}\right\|}^{2})\\ \;=-\lambda_{\min}(Q)\left[\begin{array}{cc} \left\|\mathrm{vec}(\bar{P})\right\| & \left\|\bar{x}\right\| \end{array}\right]\Theta \left[\begin{array}{c} \left\|\mathrm{vec}(\bar{P})\right\|\\ \left\|\bar{x}\right\| \end{array}\right] \end{array}$$ (22)

where $\Theta =\left[\begin{array}{cc} 1 & -\frac{L\left\|M\right\|+hc\left\|K\right\|}{\lambda_{\min}(Q)}\\ -\frac{L\left\|M\right\|+hc\left\|K\right\|}{\lambda_{\min}(Q)} & \frac{\lambda_{\min}(\bar{Q})-2c\delta \left\|KP^{*}\right\|}{\lambda_{\min}(Q)} \end{array}\right]$.

From (22), when the constant matrix Θ is positive definition, that is, the inequality $\lambda_{\min}(Q)[\lambda_{\min}(\bar{Q})‑2c\delta \left\|KP^{*}\right\|]-{\left(L\left\|M\right\|+hc\left\|K\right\|\right)}^{2}>0$ hold, then $\dot{V}$ is negative definition. Therefore, we can get that $\bar{x}$ and $\bar{P}$ are bounded, and $\bar{x}\overset{t\to +\infty }{\to }0$ and $\bar{P}\overset{t\to +\infty }{\to }0$, that is, $x\overset{t\to +\infty }{\to }x^{*}$ and $P\overset{t\to +\infty }{\to }P^{*}$. This means that the complex dynamical network composed of the subsystems (3) and (4) is asymptotical structural balance. This completes the proof of Theorem 1.

**Remark 7**. Theorem 1 shows that the nodes subsystem converges to its equilibrium point when the complex network achieves the asymptotical structural balance. This implies that the overall network comes into balance. From a social point of view, the result may be explained as the connection relationships achieve asymptotical structural balance when all individuals come asymptotically into stability in the sense of Lyapunov stability.

## Simulation example

We consider an undirected fully connected complex dynamical network consists of 10 neurons (nodes), and the dynamical behavior of each neuron is described as the dynamical model of neuron self-excitation without delays in [36]. The dynamical equations of the nodes read as

$$\dot{x}=f(x)+cP(t)H(x)$$ (23)

The dynamical equation of the connection relationships subsystem is chosen as

$$\dot{P}=AP+PA^{T}-A(xy^{T}+yx^{T})-(xy^{T}+yx^{T})A$$ (24)

where:

*f*(*x*) = [*f*_1_(*x*_1_) *f*_2_(*x*_2_) ⋯ *f*_10_(*x*_10_)]^*T*^, *f*_*i*_(*x*_*i*_) = −*x*_*i*_+(0.25+0.05*i*)tanh(*x*_*i*_−(−1)*^q^i*); *H*(*x*) = [*H*_1_(*x*_1_) *H*_2_(*x*_2_) ⋯ *H*_10_(*x*_10_)]^*T*^, *H*_*i*_(*x*_i_) = 0.1sin(*x*_*i*_). *q* is a positive integer.

Choose the parameters of simulation as follows: the common connection relationship strength *c* = 10^−6^; the constant matrix *A* ∈ *R*^10×10^ can be generated by the following rules in Matlable: (i) The matrix *A* is randomly generated with its each element to be an integer in the range [−1,1]; (ii) The matrix *A* generated in Step (i) must be a Hurwitz matrix, or repeat Step (i).

When *q* = *i*, *i*=1,⋯,10, we can obtain the following Jacobian matrix

$$\begin{array}{l} \;J=diag(-3*{\left(\tanh(x_{1}+1)\right)}^{2}/10-7/10,-7*{\left(\tanh(x_{2}-2)\right)}^{2}/20-13/20,\\ \;-2*{\left(\tanh(x_{3}+3)\right)}^{2}/5-3/5,-9*{\left(\tanh(x_{4}-4)\right)}^{2}/20-11/20,-{\left(\tanh(x_{5}+5)\right)}^{2}/2-1/2,\\ -11*{\left(\tanh(x_{6}-6)\right)}^{2}/20-9/20,-3*{\left(\tanh(x_{7}+7)\right)}^{2}/5-2/5,-13*{\left(\tanh(x_{8}-8)\right)}^{2}/20-7/20,\\ -7*{\left(\tanh(x_{9}+9)\right)}^{2}/10-3/10,-3*{\left(\tanh(x_{10}-10)\right)}^{2}/4-1/4]. \end{array}$$

When *q* = 2*i*−1,*i* = 1,⋯,10, we can also obtain the following Jacobian matrix

$$\begin{array}{l} \;J=diag(-3*{\left(\tanh(x_{1}+1)\right)}^{2}/10-7/10,-7*{\left(\tanh(x_{2}+2)\right)}^{2}/20-13/20,\\ \;-2*{\left(\tanh(x_{3}+3)\right)}^{2}/5-3/5,-9*{\left(\tanh(x_{4}+4)\right)}^{2}/20-11/20,-{\left(\tanh(x_{5}+5)\right)}^{2}/2-1/2,\\ -11*{\left(\tanh(x_{6}+6)\right)}^{2}/20-9/20,-3*{\left(\tanh(x_{7}+7)\right)}^{2}/5-2/5,-13*{\left(\tanh(x_{8}+8)\right)}^{2}/20-7/20,\\ -7*{\left(\tanh(x_{9}+9)\right)}^{2}/10-3/10,-3*{\left(\tanh(x_{10}+10)\right)}^{2}/4-1/4] \end{array}$$

Obviously, the above matrices *J* are negative definition. Therefore, there exist positive matrices $\bar{Q}$ and *K* satisfying (19).

According to the coupling matrix *Φ*(*x*), the simulation is divided into two cases.

**Case 1:** *y* = *x*

**Fig 1:**
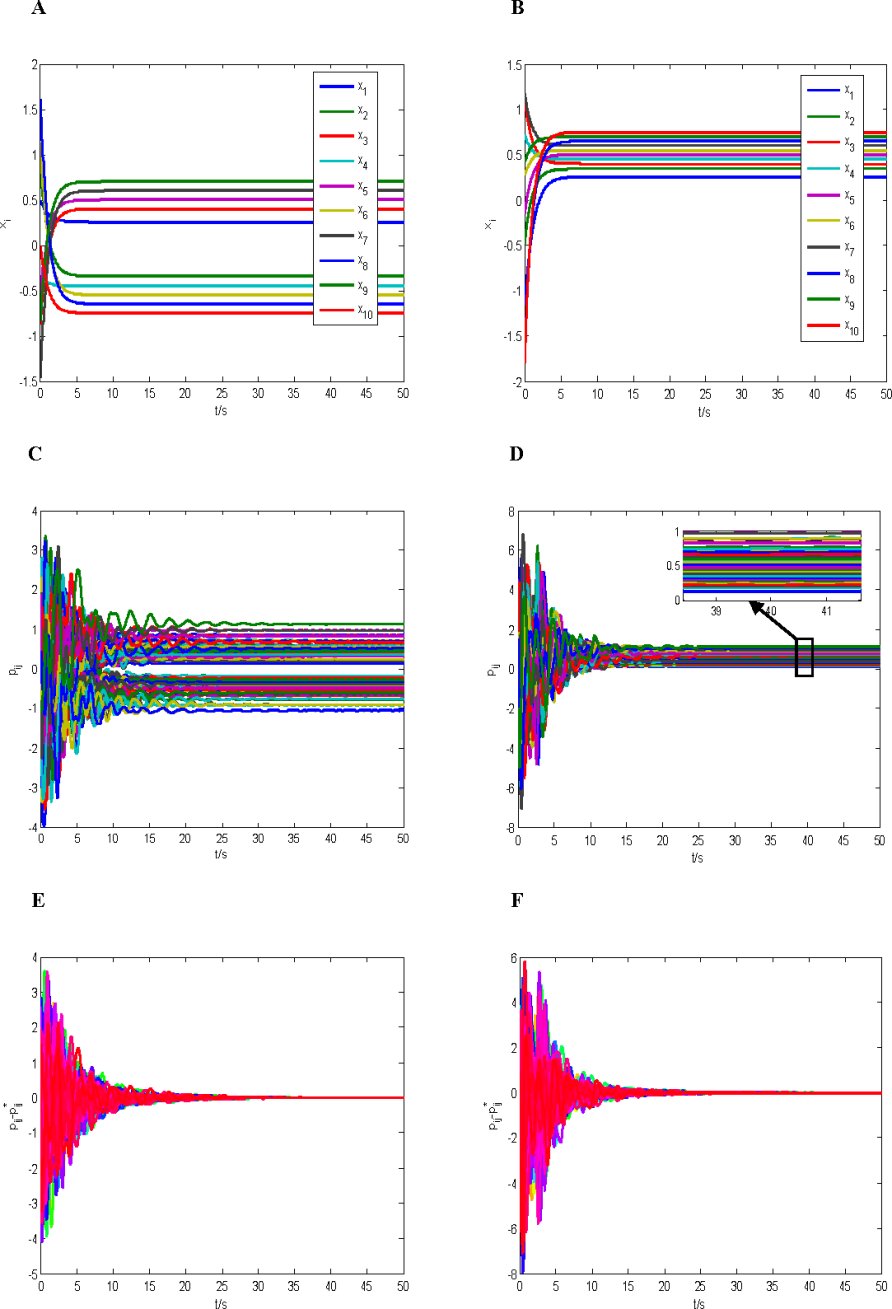
The simulation results of the case 1 (*y* = *x*). (A) The response curves of nodes group (*q* = *i*). (B) The response curves of nodes group (*q* = 2*i*−1). (C) The response curves of *P*(*t*)(*q* = *i*). (D) The response curves of *P*(*t*)(*q* = 2*i*−1). (E) The error response curves of $\bar{P}(q=i)$. (F) The error response curves of $\bar{P}(q=2i-1)$.

The universe of discourse $W=\prod_{k=1}^{10}U_{k}$, *U*_*k*_ = [−2,2], and we choose *Q* = *I* and assume *K* = 10^11^*I*, then we can obtain *h* = 0.32, *δ* = 0.32, $L=29.7\left\|\tilde{A}\right\|$, *λ*_min_(*Q*) = 1, $\lambda_{\min}(\bar{Q})\geq 0.5\times 10^{11}$, ‖P*‖≤80, and we can always choose a Hurwitz matrix *A* to make the inequality $\lambda_{\min}(Q)[\lambda_{\min}(\bar{Q})-2c\delta \left\|KP^{*}\right\|]-{\left(L\left\|M\right\|+hc\left\|K\right\|\right)}^{2}>0$ hold. Finally, initial values of states *x*_*i*_(0) and *p*_*ij*_(0),*i*, *j* = 1,2,⋯,10 are chosen as the random numbers in the range (−2,2). Fig 1 shows the results of simulation which divided into two cases of *q* = *i* and *q* = 2*i*−1.

**Case 2.** $y={\left[\eta_{1}sign(x_{1}^{*}),\eta_{2}sign(x_{2}^{*}),\cdots ,\eta_{10}sign(x_{10}^{*})\right]}^{T}$, where *η*_*i*_,*i* = 1,⋯,10 are the random number generated in the range (0, 5).

**Fig 2:**
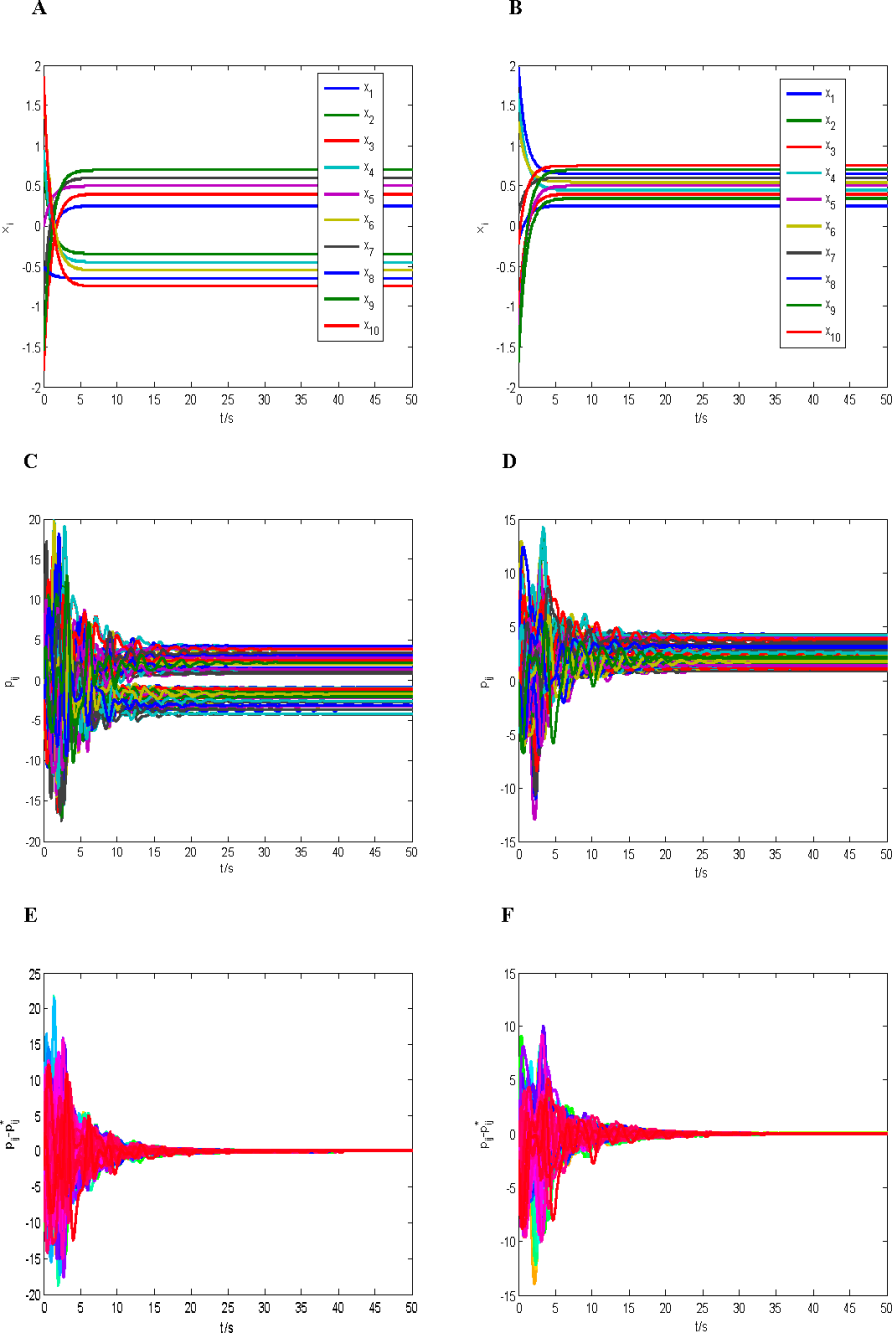
The simulation results of the case 2 $(y={\left[\eta_{1}sign(x_{1}^{*}),\eta_{2}sign(x_{2}^{*}),\cdots ,\eta_{10}sign(x_{10}^{*})\right]}^{T}$, where *η*_*i*_,*i* = 1,⋯,10 are the random number generated in the range (0, 5)). (A) The response curves of nodes group (*q* = *i*). (B) The response curves of nodes group (*q* = 2*i*−1). (C) The response curves of *P*(*t*)(*q* = *i*). (D) The response curves of *P*(*t*)(*q* = 2*i*−1). (E) The error response curves of $\bar{P}(q=i)$. (F) The error response curves of $\bar{P}(q=2i-1)$.

The universes of discourse $W=\prod_{k=1}^{10}U_{k}$, *U*_*k*_ = [−2,2] and $\bar{W}=\prod_{k=1}^{10}{\bar{U}}_{k}$, ${\bar{U}}_{k}=(0,5)$. We choose *Q* = *I* and assume *K* = 10^11^*I*, then we can obtain *h* = 0.32, *δ* = 0.32, $L=74.2\left\|\tilde{A}\right\|$, *λ*_min_(*Q*) = 1, $\lambda_{\min}(\bar{Q})\geq 0.5\times 10^{11}$, ‖P*‖<200, and we can also always choose a Hurwitz matrix *A* to make the inequality $\lambda_{\min}(Q)[\lambda_{\min}(\bar{Q})‑2c\delta \left\|KP^{*}\right\|]-{\left(L\left\|M\right\|+hc\left\|K\right\|\right)}^{2}>0$ hold. Finally, initial values of states *x*_*i*_(0) and *p*_*ij*_(0),*i*, *j* = 1,2⋯,10 are chosen as the random numbers in the range (−2,2). Fig 2 shows the results of simulation which divided into two cases of *q* = *i* and *q* = 2*i*−1.

From Figs 1 and 2, we can obtain the following conclusions:

- The complex dynamical network with neurons (23) and connection relations (24) is asymptotical structural balance.
- From Figs 1C and 2C, we can see that the connection relations between the neurons are divided asymptotically into two parts with the dynamical changes of internal state of the neurons and the coupling matrix. This implies that all neurons are partitioned asymptotically into two factions, such that all links between neurons of the same faction are positive and all links of the two different factions are negative. Finally, the structural balance of the network is achieved. It is also observed from Figs 1D and 2D that the connection relations between the neurons are all positive, it means that all neurons are in the same faction. This kind of network is called as the trivial structural balance. The above observed results coincide in the ones in [3,6,8]. It is noted that the similar results is not possible for some models in earlier literature, because these models contain so-called jammed states that could trap a social network before it reached a two-faction configuration [37,38].

In this paper, there are three reasons to result in the above conclusion (ii): **(a)** The form of P* is determined by the form of coupling matrix *Φ*(*x*) shown in (8). That is, if the equilibrium state {x*,P*} exists, then we can obtain P* = x*y^T^ + *y*(x*)^*T*^; **(b)** If *q* = *i*, the signs of the equilibrium points in the subsystem (23) are different with positive and negative ones, and thus there must be existing both positive and negative elements in the matrix P*, which leads to the connection relationships are divided asymptotically into the two factions. If *q* = 2*i*−1, there only exist positive equilibrium points in the subsystem (23), and thus each element of the matrix P* is positive, which leads to the connection relationships form only one faction. **(c)** If *q* = *i*, the numbers of positive and negative equilibrium points are equal in the subsystem (23), and thus the numbers of positive and negative elements in the matrix P* are also equal. This implies that the two factions divided have the same members. A more general form is given here, if the coupling matrix *Φ*(*x*) is shown in (8), and the Eqs (5) and (6) hold, we assume that the number of the positive equilibrium points is $\bar{N}(\bar{N}\leq N)$ in the subsystem (3), then we obtain the number of the positive elements in matrix P* is ${\bar{N}}^{2}+{\left(N-\bar{N}\right)}^{2}$, and the number of the negative elements is $2\bar{N}(N-\bar{N})$. Obviously, ${\bar{N}}^{2}+{\left(N-\bar{N}\right)}^{2}\geq 2\bar{N}(N-\bar{N})$, the equality is true if and only if $\bar{N}=\frac{N}{2}$. This shows also that the numbers of the curves is the same in the two factions in Figs 1C and 2C.

## Conclusion

In this paper, we have analyzed and proved the asymptotical structural balance for a class of complex dynamical networks by designing the coupling matrix under some conditions, that is, we mainly pay attention to the dynamic mechanism by which the nodes act as the connection relationships subsystem to generating the structural balance. We presented mathematically the dynamic models for the nodes and connection relationships subsystems in which their equilibrium points exist. It is found in this paper that the coupling matrix in the connection relationships equation plays an important role in the structural balance to be achieved. The links with positive and negative values may make the connection relationships to be divided asymptotically into the two factions, which is explained by using a network with neurons. These links in the network evolve according to the nodes’ dynamics, in the sense of society, which reflect an individual desire to drive the social relations into the structural balance via personal behavior. Therefore, this method proposed in this paper makes up the deficiencies of researches methods on the structural balance theory in social networks. This enriches the research contents of complex dynamical networks and structural balance theory.

## Supporting information

S1 Text A Hurwitz matrix *A* is given which satisfies the condition. (PDF)
