# Supplementary material for: The structural balance analysis of complex dynamical networks based on nodes' dynamical couplings
Source: PLoS One. 2018 Jan 31;13(1):e0191941. doi: 10.1371/journal.pone.0191941 (PMC5792007; doi:10.1371/journal.pone.0191941)
Supplement: S1 Text — (PDF) [file pone.0191941.s001.pdf]

A Hurwitz matrix A is given which satisfies the condition

In this paper, we can choose a Hurwitz matrix

$$A=\begin{bmatrix} -1 & 0 & 1 & 0 & 0 & 1 & 1 & -1 & -1 & 0 \\ 0 & 0 & -1 & 1 & 0 & -1 & 1 & 1 & 0 & 0 \\ -1 & 1 & -1 & 0 & 0 & 1 & 0 & 1 & 0 & 0 \\ -1 & -1 & 0 & -1 & 1 & 0 & 1 & -1 & 1 & -1 \\ -1 & 1 & -1 & -1 & 0 & -1 & -1 & 1 & 1 & 0 \\ -1 & 1 & 1 & 1 & 1 & -1 & -1 & 1 & -1 & 1 \\ 0 & -1 & -1 & -1 & 1 & 0 & -1 & 1 & 1 & 1 \\ 0 & 1 & -1 & 0 & -1 & -1 & -1 & -1 & -1 & 0 \\ 1 & 0 & 1 & -1 & 1 & 1 & -1 & 0 & -1 & 1 \\ 1 & 0 & -1 & 1 & 0 & 0 & -1 & 1 & 0 & 0 \end{bmatrix}$$ in the simulation.

According to the coupling matrix $\Phi(x)$, the condition is divided into two cases, and we need to make sure that the condition is established in every case.

Case 1: $y = x$

We choose $Q = I$ and assume $K = 10^{11} I$, then we can obtain $h = 0.32$, $\delta = 0.32$, $L = 29.7\|\tilde{A}\|$, $\|\tilde{A}\| = 38.7$, $\lambda_{\min}(Q) = 1$, $\lambda_{\min}(\bar{Q}) \geq 0.5\times 10^{11}$, $\|P^*\| \leq 80$, $\|M\| = 65.77$.

That is $\lambda_{\min}(Q)[\lambda_{\min}(\bar{Q}) - 2c\delta\|KP^*\|] - (L\|\mathrm{M}\| + hc\|K\|)^2 \geq \lambda_{\min}(Q)[\lambda_{\min}(\bar{Q}) - 2c\delta\|K\|\|P^*\|]$ $-(L\|\mathrm{M}\| + hc\|K\|)^2 \geq 0.5\times 10^{11} - 2\times 10^{-6}\times 0.32\times 3.16\times 10^{11}\times 80 - (29.7\times 38.7\times 65.77 +$ $0.32\times 10^{-6}\times 3.16\times 10^{11})^2 > 0$ hold.

Case 2. $y = \left[\eta_1 sign(x_1^*), \eta_2 sign(x_2^*), \cdots, \eta_{10} sign(x_{10}^*)\right]^T$, where $\eta_i, i = 1, \cdots, 10$ are the random number generated in the range (0, 5).

We choose $Q = I$ and assume $K = 10^{11} I$, then we can obtain $h = 0.32$, $\delta = 0.32$, $L = 74.2\|\tilde{A}\|$, $\|\tilde{A}\| = 38.7$, $\lambda_{\min}(Q) = 1$, $\lambda_{\min}(\bar{Q}) \geq 0.5\times 10^{9}$, $\|P^*\| < 200$, $\|M\| = 65.77$.

That is $\lambda_{\min}(Q)[\lambda_{\min}(\bar{Q}) - 2c\delta\|KP^*\|] - (L\|\mathrm{M}\| + hc\|K\|)^2 \geq \lambda_{\min}(Q)[\lambda_{\min}(\bar{Q}) - 2c\delta\|K\|\|P^*\|]$ $-(L\|\mathrm{M}\| + hc\|K\|)^2 > 0.5\times 10^{11} - 2\times 10^{-6}\times 0.32\times 3.16\times 10^{11}\times 200 - (29.7\times 38.7$ $\times 65.77 + 0.32\times 10^{-6}\times 3.16\times 10^{11})^2 > 0$ hold.
